# A Quantitative Systems Pharmacology Perspective on the Importance of Parameter Identifiability

**DOI:** 10.1007/s11538-021-00982-5

**Published:** 2022-02-07

**Authors:** Anna Sher, Steven A. Niederer, Gary R. Mirams, Anna Kirpichnikova, Richard Allen, Pras Pathmanathan, David J. Gavaghan, Piet H. van der Graaf, Denis Noble

**Affiliations:** 1 Pfizer Worldwide Research, Development and Medical, Massachusetts, USA; 2 King’s College London, London, UK; 3 Centre for Mathematical Medicine and Biology, Mathematical Sciences, University of Nottingham, Nottingham, UK; 4 University of Stirling, Stirling, UK; 5 Center for Devices and Radiological Health, U.S. Food and Drug Administration, Maryland, USA; 6 Department of Computer Science, University of Oxford, Oxford, UK; 7 Certara QSP, Canterbury, UK; 8 LACDR, University of Leiden, Leiden, The Netherlands; 9 Department of Physiology, Anatomy and Genetics, University of Oxford, Oxford, UK

**Keywords:** Quantitative systems pharmacology, Model identifiability, Model development

## Abstract

There is an inherent tension in Quantitative Systems Pharmacology (QSP) between the need to incorporate mathematical descriptions of complex physiology and drug targets with the necessity of developing robust, predictive and well-constrained models. In addition to this, there is no “gold standard” for model development and assessment in QSP. Moreover, there can be confusion over terminology such as model and parameter identifiability; complex and simple models; virtual populations; and other concepts, which leads to potential miscommunication and misapplication of methodologies within modeling communities, both the QSP community and related disciplines. This perspective article highlights the pros and cons of using simple (often identifiable) vs. complex (more physiologically detailed but often non-identifiable) models, as well as aspects of parameter identifiability, sensitivity and inference methodologies for model development and analysis. The paper distills the central themes of the issue of identifiability and optimal model size and discusses open challenges.

## QSP and PK/PD

In the last one hundred years, pharmaceutical drug discovery and development can be characterized by a trend toward both mechanism-driven discovery and a more quantitative approach to development, efficacy and safety assessment. These trends were a result of advances in molecular biology, biochemistry and genetics. In particular, the shift toward quantitative approaches in pharmaceutical R&D was reflected in the regulatory guidelines introduced in the 1970s that advocated the use of Pharmacokinetics (PK) modeling in drug and trial design. Subsequently, in recent decades, advances in mathematical modeling, evolution of computational power and software, and accessibility of large preclinical and clinical data sets have contributed to the emergence and establishment of the Quantitative Systems Pharmacology (QSP) field. QSP is a relatively new discipline that integrates Pharmacokinetics/Pharmacodynamics (PK/PD) and Systems modeling approaches. QSP was formalized as a research area around 2011 in a white paper (Sorger et al. 2011) published by the US National Institutes of Health. It combines biophysically detailed mechanistic models of physiology in health and disease with PK/PD to predict systemic effects. Applications of QSP range from generating and exploring new mechanistic hypotheses of an observed effect, identifying optimal or alternative targets, gaining confidence in rationale of existing and/or emerging targets, designing preclinical and clinical experiments and clinical trials, providing insight from preclinical to clinical translation or cross-disease translation (for instance, in the case of drug repurposing).

Originally developed as a research tool, PK/PD was adapted by the pharmaceutical industry to address a central need in the drug development pipeline by providing a formal framework for predicting dosing regimens in early stage clinical trials. The development of best practices and the formalization of PK/PD modeling through regulatory guidelines cemented the PK/PD approach as a crucial component of any drug development program. The development of QSP is currently following a similar pattern. As adoption of QSP increases (Musante et al. 2017; Zineh 2019), it is expected to become an integral part of regulatory requirements in the drug development and approval process [e.g., the Comprehensive In silico Pro-arrhythmia Assay (CIPA) Initiative (Li et al. 2018)]. With the rapidly growing use of QSP modeling in basic, preclinical and clinical research, there is a mounting interest in identifying best practices, techniques and open challenges in QSP methodology and tools (Ribba et al. 2017). These challenges include selection of appropriate models to work with, efficient parameter estimation, examination of parameter identifiability, incorporation of virtual population studies, application of sensitivity analysis and model reduction techniques, as well as validation, verification and uncertainty quantification (VVUQ) (Pathmanathan and Gray 2013). As QSP transitions from a research area to a drug development tool, also recognized by regulatory agencies, it is important to develop and agree upon best practices to ensure successful application of QSP modeling in drug discovery, design and development (Bai et al. 2019). At present, regulatory agencies, industrial and academic experts are working together to put forward standards that could be adopted for regulatory purposes when assessing credibility and validity of a QSP model.

## QSP and Identifiable and Non-identifiable Models

The major motivation for QSP models is that our knowledge of biology and pharmacology is increasingly too complex for intuition-based analyses. Mathematical biologists and computational modelers strive to build mathematical models in order to understand the biological and physiological mechanisms underlying the system’s behavior. Only by encoding our knowledge in mathematical models to quantitatively represent the system under study can we hope to understand emergent biological behavior, its regulation by underlying mechanisms, how these mechanisms are compromised by pathologies and whether they can be manipulated pharmaceutically. Emergent behavior can arise from small or large models, however representing complex physiology often requires developing mathematical models of large biological systems with a correspondingly large set of unknown model structures and parameters, which may result in non-identifiable models (due to overparameterization and limited data).

What do we mean by ‘identifiability’? First of all, the terms model identifiability, parameter identifiability, and model parameter identifiability are considered to be interchangeable for the purpose of this paper, as well as, to the best of our knowledge, by the QSP modeling and related disciplines. Similarly, the term ‘non-identifiable’ is used interchangeably with ‘unidentifiable’ by the research community subject to personal preference of the author. Identifiability analysis approaches, overviewed recently in a number of works (including in Raue et al. 2009, Saccomani et al. 2013), can be categorized into structural and practical non-identifiability. Structural non-identifiability (Cobelli and DiStefano1980) is related to the model structure and whether each model parameter can have an independent effect on the observed model output. A trivial example of structurally non-identifiable model is the model *y* = *ab*x where it is not possible to uniquely identify parameters *a* and *b* given measurements of (*x,y*). For more complex models (e.g., systems of ODEs), structural identifiability can be more difficult to determine, but a variety of analytical and numerical methods are available to assess structural identifiability (Chis et al. 2011; Miao et al. 2011; Kreutz 2018; Karlsson et al. 2012). Practical non-identifiability considers whether the amount and quality of a particular experimental dataset used for parameter calibration constrains parameter estimates (Raue et al. 2009; Shotwell and Gray 2016). Practical identifiability implies structural identifiability; however, the reverse is not true. The difference between structural and practical identifiability analysis can be summarized as following: structural identifiability analysis is a mathematical exercise that asks if parameters are unique given hypothetical perfect noise-free data, whereas practical identifiability involves analyzing the data available. In general, a QSP model can be classified into three major categories: (i) models that are provably structurally identifiable and are also practically identifiable given the data (these are typically simpler QSP models, though we emphasize that simple models need not be structurally identifiable); (ii) models that are probably or provably structurally identifiable but not practically identifiable given the data available; and (iii) models that are provably or probably structurally non-identifiable, and therefore not/not expected to be practically identifiable, regardless of data quality. Many complex QSP models will fall in the last category. The below discussion is relevant for QSP models in groups (ii) and (iii). Below we focus primarily on practical identifiability, and from here onward ‘identifiable’ refers to structurally and practically identifiable, and ‘non-identifiable’ refers to practically non-identifiable (may or may not be structurally identifiable). However, some of the below discussion, e.g., Sect. 5, will also be relevant to structurally non-identifiable QSP models.

What do we mean by ‘identifiable’ model parameters? Ideally, we might mean a point estimate can be given for each parameter after fitting to some data. But practically, given noise and other sources of variability, we mean a ‘constrained’ (a subjective term) probability distribution for the set of parameter values around a point (ideally including covariance by fitting a single probability distribution across all parameters at once). A model is non-identifiable if calibrating using available noisy data does not permit a constrained probability distribution or bound for the parameter value. Non-identifiable parameters will not be constrained and could take a wide range of values, often covarying with other parameters such that groupings (sums or products of parameters, or more complex model outputs) are constrained but the individual parameters are not.

It is important to keep in mind in the discussion below that there is no such thing as a practically ‘identifiable model’ or ‘identifiable parameter’—this is just shorthand for a ‘parameter of a given model is identifiable given the data this model structure was fitted to’. A hypothetical perfect experiment could measure any parameter (rate constant, concentration, etc.), and efforts should be made to optimize experiments to attempt to do this. Identifiability is not relevant to parameters that can be directly measured or inferred by other means, rather than estimated through model calibration. It was suggested that it may, therefore, be better to talk about ‘unidentified’ parameters, reserving the term ‘unidentifiable’ for structural or a priori unidentifiability (Fink and Noble 2009). However, this terminology has not been widely adopted by the research community.

When designing a model, we are often faced with a tradeoff: build a simpler model with more identifiable parameters, but which may not be able to capture multiple mechanisms (which could be crucial for identifying novel drug targets) and might sacrifice accuracy compared to the data for its simplicity, or build a more complex model, for which we will have difficulty choosing the right parameters. There are methods to mitigate the disadvantages of each option, such as model discrepancy methods for simple models and virtual populations and uncertainty propagation for non-identifiable models. The research field is rife with arguments about when each kind of model is appropriate. In particular, the proliferation of non-identifiable models has recently led to discussions on the appropriateness of their use. Indeed, the suitability of non-identifiable models for prediction has been an ongoing debate in the mathematical modeling and more recently in the QSP community, with many emphasizing the benefits of identifiable models (Munoz-Tamayo et al. 2018). The proponents of identifiable models argue that with complex models, overfitting is unavoidable, calling into question the utility of complex models overall. One of the practical questions for the QSP community is whether we as a community are better off dismissing non-identifiable models altogether.

We suggest that an important component to this discussion is the proposed utility of the model. Broadly, model uses can be classified as interpolative (for example, predicting response for intermediate doses or time-points) or extrapolative (longer time-points, higher doses, different dose regimens, predicting novel drug combinations). For many extrapolative use cases, such as predicting the effects of novel drug combinations, a more complex model (likely non-identifiable) could be necessary. Therefore, if one wants to argue to dismiss non-identifiable models altogether, a corollary is a large reduction in the number of applications QSP models can support. While this may or may not be technically appropriate, it is worthwhile to note that the application of the “mental models” of biologists/clinicians that QSP aims to formalize are not typically restricted in scope.

The objective of this perspective article is to discuss the rationale for building and using identifiable versus non-identifiable models, as well as to highlight techniques that reduce large models, make models ‘simpler’ and identifiable, and quantify model uncertainty especially relevant in building confidence when applying non-identifiable models.

## Model Development and Complexity

The usual modeling process consists of (1) model development through training or calibration (where model structure and model parameters are derived based on experimental data and hypotheses of the underlying system’s behavior), (2) model validation or testing (where model outputs are evaluated against experimental data not used at the calibration stage), followed by (3) model predictions.

To make sense of inherent complexity of nature, it is often helpful to start by simplifying and partitioning a complex biological system and using simple or phenomenological models to describe the underlying mechanisms and resulting phenomena (as Occam’s razor would suggest). The resulting individual models are typically identifiable if appropriate training data and parameter estimation techniques are used.

As we develop and improve models, there is a tendency to describe biological and physiological processes in more detail and hence generate more complex models. Such models are difficult to make identifiable for a few reasons. First, experimental data can be lacking or may insufficiently discriminate between different parameter settings. Moreover, experimental data sets describing the same phenomenon from different research laboratories may differ and hence result in limited reproducibility (e.g., Niepal et al. 2019; Hirsch and Schildknecht 2019). In addition, it is more difficult to confirm uniqueness of optimal parameter settings in complex models due to the high dimensionality of the parameter space. Also, mechanistic models are sometimes non-identifiable due to the existing tendency in the field to combine different smaller identifiable models (each representing, for instance, a particular compartment or pathway inside a cell) without re-parameterizing the newly combined model using all of the previous experimental training sets from the smaller models. In particular, the task of building models of multiple interacting components (e.g., proteins) or systems by one individual, or even one research group, is laborious and sometimes intractable which often leads to model reuse, the coupling of incompatible pre-existing models which may represent different species and/or incompatible conditions (e.g., temperature, cell type (Niederer et al. 2009)), further obfuscating the link between model parameters and experimental data, again leading to non-identifiability. In addition, when performing optimization, often training experimental data does not include variation of initial conditions which may be required to constrain the parameters of the model in cases of multistability in biological systems (Surovyatkina et al. 2010).

From an evolutionary perspective, physiological and biological redundancy is inherent to biological systems often protecting against the impairment of certain functions that are vital for the survival of an organism. Examples of functional redundancy in nature exist at every level from gene to protein to cell to organism. For instance, consider the genetic compensation for the altered function of certain proteins (Giaever and Nislow 2014; Roden 2008), or the pacemaker cells of the heart (sino-atrial node cells, atrio-ventricular node cells, Purkinje fibers) that send out electrical signals to activate cardiac muscle contraction but do it at different frequencies, hence providing a safety mechanism. Capturing such compensation mechanisms in mathematical models can naturally lead to non-identifiability, as experiments may have trouble distinguishing between the primary mechanism and the compensation. Building models that capture physiological redundancy and yet are identifiable requires special care in collecting training experimental data, especially as we do not always know in advance that redundant regulation may be involved.

Often, it is not feasible to develop an identifiable model for a system with numerous redundant mechanisms since one cannot provide detailed experiments (e.g., knock out each potential mechanism to constrain parameters) due to limited time and resources, and, in addition, generation of such detailed experimental data may defeat one purpose of modeling which is to provide a tool for evaluating different hypotheses, predicting behavior under new conditions and suggesting additional experiments for rejecting hypotheses. From this point of view, given the reality of limited biological a priori knowledge of the underlying mechanism of action and sparse/limited data, a non-identifiable model that is adequately validated (and context-appropriate) could be argued to be fit-for-purpose, despite not being identifiable. Such a model is believed to be useful for revealing missing mechanisms and a tool for gaining confidence in mechanism of action. The confidence is gained through prediction of anticipated effects of existing mechanisms and due to a constant ‘model development → experimental validation success → experimental prediction failure → model improvement’ cycle.

Others argue that a mathematical model has predictive power only if it is identifiable (e.g., Beattie et al. 2013; Whittaker et al. 2020), because non-identifiable, over-parameterized models (while reproducing datasets they are trained and validated against) may yield misleading results and conclusions, especially when predicting responses under new conditions different to the validation conditions (Lei et al.)—which is often the goal of the mathematical modeling.

## Identifiable Models: Why Should One Worry about Model Non-identifiability and What to do if the Model has Too Many Parameters?

In the case of identifiable models, the distribution of possible model output dynamics constrained by experimental data yields tightly constrained input parameters. In the case of non-identifiable models, the model may perform well and may give constrained predictions in select new settings. However, certain new model dynamics behavior in a non-identifiable model may depend strongly on the unconstrained parameters, leading to potentially misleading results. As stressed by Mirams and others, over-parameterized models can reproduce the datasets they are calibrated against but are often unable to predict new regimes of biological phenomena due to their non-identifiable parameters (Beattie et al. 2013).

To illustrate, Mirams proposes to consider a scenario of how a non-identifiable model might make sensible predictions when used in a dynamical regime close to where it was developed, calibrated and/or trained, but lead to a wide and potentially unconstrained range of predictions in situations away from this. Such a situation may be provoked, for instance, by the activation or blocking of a reaction, or a change of boundary or initial conditions. Mirams’ research shows that in regimes away from the calibration regime, an overly and unrealistically wide range of possible model outputs may be produced by non-identifiable models (Fink et al. 2011; Whittaker et al. 2020), and this has nothing to do with biological variability—it is purely a product of lack of knowledge about parameter values. The different sources of uncertainty in parameter values are important to distinguish and are referred to as *aleatory* (‘irreducible’ uncertainty, e.g., arising from natural biological variability) and *epistemic* (‘reducible’ uncertainty, arising from lack of knowledge) uncertainty (Mirams et al. 2016). Uncertainty propagation should therefore be performed to determine the regime before using predictions from models with non-identifiable parameters. Observing particularly large uncertainty in model behavior suggests that a newly predicted output set contains model outputs that are sensitive to the non-identifiable parameter(s), and additional experiments that determine the true value of the model outputs could then be used to re-train the model and identify the previously ‘non-identifiable’ parameters.

Importantly, one problem is that if simply ‘best fit’ point estimates are used for parameter values, it can be difficult to determine whether our parameters are constrained or not. Mirams advocates using inference techniques to derive probability distributions for parameters, where it is immediately evident whether parameters’ values are constrained (Siekmann et al., 2012). Subsequent predictions should be made using Uncertainty Propagation (Pathmanathan and Gray 2013) and, if it is a feature of the system, the unconstrained behavior due to non-identifiable parameters will become evident. These are the fundamentals of Verification, Validation and Uncertainty Quantification (National Academies 2012). As highlighted by Mirams, if just point estimates are made with an unidentifiable model, one will have no way of knowing where in the region of possible predictions (plausible or non-plausible) one lies, or how large the region of equally-plausible behavior is.

Mathematical, computational, physical and engineering fields other than QSP have been faced with the dilemma of non-identifiable models, and thus an array of tools exists to help tackle practical questions including ‘how to know and how to test if a model is too big?’, ‘how to test if the model is identifiable’, ‘how to reduce a model?’. For example, the review by Snowden (Snowden et al. 2017) summarizes model reduction methods including time scale exploitation, truncation and lumping (e.g., Gulati et al. 2014; Hasegawa et al. 2018). The structural and practical identifiability analyses (Raue 2009; Raue 2014) of models by exploiting the profile likelihood method and helping reduce complex models have been increasingly employed both in application to PK/PD including cardiac safety investigations (Cheung et al. 2011) and QSP including modeling erythropoietin receptor (Becker et al. 2010) and JAK2/STAT5 signaling (Bachmann et al. 2011). Sensitivity analysis is another tool that can be employed to evaluate parameter significance and inform model reduction (Saltelli et al. 2008). The listed techniques are examples and not a comprehensive review of methods and tools available.

Successful application of phenomenological, ‘simple’ models span many disease areas including oncology, neuroscience, immunology, cardiovascular (Gray and Pathmanathan 2016). Moreover, as highlighted by Mistry et al. 2015, Mistry 2018 and Parikh et al. 2019 in the case of Torsades de Pointes drug safety prediction studies, simple models and linear regression analyses can perform as well as or better than complex models. These works highlight that complex approaches bring additional computational cost, increased noise and increased error in predicted behaviors, yet do not necessarily translate into additional understanding of the underlying mechanisms.

Given that a large number of existing QSP models are complex and non-identifiable, let us consider situations when it could be appropriate to use complex models, and what the objective of using complex models could be.

## Non-identifiable Models: When is it Appropriate to Use Poorly Constrained but Physiologically Rich Models?

Undoubtedly the ability to accurately predict therapeutic or toxic effects of novel compounds in a cell, organ or an entire organism in animals and in humans using in silico tools would have a dramatic impact on drug discovery and development. To achieve this goal will require well-constrained mathematical models that provide a sufficiently detailed representation of the underlying physiology to make useful predictions (Ribba et al. 2017). While this is the long-term goal of quantitative systems pharmacology, and specific examples do exist (Beattie et al. 2013; Mirams et al. 2011), in the general sense this is not currently possible. Many models are poorly constrained (Gutenkunst et al. 2007), often due to structural unidentifiability (Cheung et al. 2011) and/or a scarcity of relevant data. The result is that many large complex models reuse existing models that are not constrained to the specific setting (Fink et al. 2011; Niederer et al. 2009), implicitly assuming that inter-species differences, temperature dependence, cell type and experimental protocol have a limited impact on the model prediction of interest. While these assumptions do pose significant limitations on making quantitative predictions, the complex models do provide a physiologically motivated and physically constrained framework for making qualitative estimates of the effect of a novel compound on a physiological system and have a role to play in quantitative systems pharmacology (e.g., Guyton and Coleman 1969; Peterson and Riggs 2010; Allen et al. 2016; Allen and Musante 2018).

There are many decisions in drug discovery and development where there is simply insufficient information to make a decision on which compounds to progress or which compounds to study first. In these use cases, assuming a detailed and highly predictive model is unavailable, a pre-existing complex unidentifiable model may prove useful. These complex models can be beneficial in specific cases. One use of complex models is for proposing potential biological mechanisms that will be indirectly affected by a novel compound. For example, a computational model could predict that a compound that alters the electrophysiology of the cell by binding to a membrane-bound electrogenic pump, such as the effect of digitalis on the sodium –potassium pump, can indirectly cause significant changes in myocardial contraction (Langer 1977). Also, complex models can be used for providing a ranking of compounds when limited information is available. Many drugs are developed from compound libraries, and lists of candidate compounds are iteratively refined during the drug development process (Smith 2002). Complex models can be used to rank candidate compounds based on a best guess of, for example the Torsade des Pointes risk (Davies et al. 2016). This is not to suggest that a model should be used to remove a candidate compound but if experiments are performed sequentially, one compound needs to be tested first and complex models provide a framework for identifying that compound. Complex models can be successfully applied when building confidence in particular mechanism of action (Tewari et al. 2016; Hallow et al. 2018), helping design preclinical and clinical experiments, translating between species or evaluating efficacy margins (Peterson and Riggs 2010). Further, importantly, if and when this process fails, the information can be fed back in to improving the complex model.

Complex models can have a number of limitations due to the potential lack of reliability of their predictions, and special techniques are necessary to provide estimates of the fidelity of the model predictions. These include the estimation of uncertainty in model parameters given the available data and how they impact model predictions. Specific examples include formal Monte Carlo Markov Chain Sampling Bayesian methods (Johnstone et al. 2016), Bayesian inference approaches to uncertainty quantification (McKinley et al. 2018; Ghanem et al. 2017), history matching ideas and ensemble studies adopted from climate sciences (Williamson et al. 2013), virtual populations generation and selection studies (Allen et al. 2016; Rieger et al. 2018), recently proposed ‘output-matching’ approaches (Britton et al. 2013; Sobie 2009), sensitivity analysis (Iooss and Lemaitre 2014; Chang et al. 2015; Saltelli et al. 2019). More recently, these ideas have been extended to how uncertainty in numerical approximations will affect parameter estimation (Oates et al. 2016). If key model predictions are dependent on well constrained parameters fitted to relevant data (even when the model includes non-identifiable parameters), this gives more confidence in the predictions. Performing model analysis (including global sensitivity) is critical and recently has been becoming more mainstream as researchers realize that the limitations and confidence in the predictions of complex models need to be assessed.

All models as well as all measurements are approximate, and we always have to keep in mind the question ‘how much error can we tolerate?’ Uncertainty quantification and careful examination of sources of error is critical to ensure accurate conclusions, whether the error is due to the experimental setup, measurement bias, reproducibility, inter-experimental variability, true underlying biological variability, numerical solution accuracy or chosen model structure.

Examples such as Allen et al. (2016) are illustrative case studies when poorly constrained but physiologically rich models provide significant insight in a timely and cheap fashion, avoiding costly experiments. The right question for the model and the right techniques of uncertainty estimation are essential components for the use of non-identifiable models. For instance, the purpose of virtual populations is that, as additional data becomes available, one is able to constrain the acceptable parameter space, moving toward a more identifiable model.

Furthermore, when designing a physiological model, encountering non-identifiability may point to a gap in biological knowledge and suggest which new measurements would improve the understanding of the system. Finally, complex models also provide a starting point for model reduction techniques (Snowden et al. 2017); however, it is important to ensure that these methods are not removing crucial model components and functions.

## Practical Challenges of Model Development and the Merit of Different Approaches

We feel that while the use of either identifiable or non-identifiable models is valid, what is important is that the users are aware of the assumptions they are making and are familiar with the notion of identifiability so that they can rightly inform their future applications of a model. The extent to which identifiability matters is also often closely interlinked with the specific question of interest and the model’s context of use. For instance, knowing a priori whether the context of use will involve ‘interpolation’ or ‘extrapolation’ can be helpful in deciding whether a large non-identifiable complex model is appropriate to employ. In the case of interpolation (i.e., making predictions of behavior close to or within the validated regimes), identifiability plays a lesser role for building confidence in the results and decrease of the uncertainty. In such cases, it is not necessarily advantageous to aim for developing and employing minimalistic, identifiable models (which, counterintuitively, may be more time consuming and challenging to build than a more complex throw-every-mechanism-into-the-bag one). In the case of extrapolation, on the other hand, model identifiability is likely to be more cost-effective by removing the need for additional iterations of the experimental cross-check, validation, model improvement via ‘model development → experiment → model improvement’ cycle. However, as noted, some extrapolative questions necessitate the incorporation of additional biological mechanisms which may lead to non-identifiability issues.

In the case of extrapolation, uncertainty quantification and the back-and-forth cycle between model and experiments has a more important role in verifying hypotheses and improving the model. For instance, generation and selection of virtual populations (Allen et al. 2016; Rieger et al. 2018) help classify results based on assumptions of model output correlations, input parameter and model output ranges, and model structure (e.g., Markov chain vs Hodgkin-Huxley formulation, reversible or irreversible reactions, etc.). Non-identifiable models may help exclude certain mechanistic hypotheses by failing to generate particular outputs (plausible or experimentally observed) in any virtual population member. Suppose that we have a model that describes a mechanistic hypothesis: if a model cannot reproduce certain behavior regardless of parameter setting, this provides ground for rejecting a hypothesis even if the model is non-identifiable. The context of model use is thus central.

The impact and future of mathematical modeling of biological systems is predicated on supporting the design and/or analysis of experimental and clinical data. To that extent, the relevant comparison is less non-identifiable vs. identifiable models but quantitative modeling support vs. human intuition. In this context, non-identifiable (or more complex) models might have advantage in engaging biologists and clinicians in the modeling process (due to typically being at a similar scope as their concept of the system), whereas identifiable models might gain support and trust from the same community in driving robust predictions. Again, we note that context is crucial—a given system and dataset requires careful consideration as to the appropriate model, and neither non-identifiable nor identifiable models should be dismissed a priori.

The ability to build, fit (i.e., calibrate) and use QSP models, while addressing challenges associated with unidentifiability, is also highly dependent on the availability of and access to high-quality experimental data. Each individual researcher or group of researchers (in academia, industry, or government agency) lacks the resources to do all experiments themselves and must rely on data from other teams. Further, in case when complex models are built on other models, one needs access to the code and data used for fitting of the previous models and experimental data used to fit those. As a result, some of the challenges of building QSP models are not mathematical, but rather practical. Examples of practical challenges include (i) transparent model code access and transparent data (used for training and validation) storage, (ii) reproducibility of model simulation results, (iii) automated model validation, (iv) knowledge transfer of negative results. These challenges remain even in well-established fields such as mathematical biology, mathematical physiology, computational biology and systems modeling.

Transparent model code access and transparent data storage is becoming increasingly important as models become larger and parameter inference becomes more complex. Further, as the QSP field develops, for QSP modeling there will be a growing regulatory expectation on model validation and verification. The gold standard for academia, regulatory agencies and industry is publishing models and associated data and code in peer reviewed journals. The issue of access to training and validation data (in the cases concerning confidential individual data or compound specific data) still remains yet sometimes this can be overcome by providing data averaged over individuals or publishing the underlying systems model without the pharmacology model. In the latter case, removing the pharmacology portion of the model may result in the model being less identifiable as part of datasets used to fit the model is unavailable. However, virtual populations of such less-identifiable published model can be used by others to constrain parameter spaces based on additional data available to them.

Ideally, in a future where significant progress has been made on the above practical challenges, we would have the time, experimental capability, and resources to gather enough data to identify all parameters even in complex models that are today considered non-identifiable. However, given the limitations of the real world, non-identifiability is a reality. Both identifiable and non-identifiable model can be advantageous to employ, and the pros and cons of model identifiability depend on the intended context of use. One thing is certain: managing and quantifying uncertainty in parameters and output features is a critical component in assessing the validity and predictive power of a model.
